# Supplementary material for: Gender differences in dictator giving: A high-power laboratory test
Source: PLoS One. 2025 Feb 12;20(2):e0317886. doi: 10.1371/journal.pone.0317886 (PMC11819478; doi:10.1371/journal.pone.0317886)
Supplement: S1 File — (DOCX) [file pone.0317886.s001.docx]

**Supporting information to**

**Gender differences in dictator giving: a high-power laboratory test**

**Iván Barreda-Tarrazona, Ainhoa Jaramillo-Gutiérrez,**

**Marina Pavan and Gerardo Sabater-Grande**

*LEE & Economics Department, Jaume I University, Castellón, Spain.*

**S1 - Experimental Instructions (translated from Spanish):**

***General Instructions***

- The purpose of this experiment is to study how individuals behave in certain economic contexts. The experiment consists of two sessions: one, carried out today, and another one, next week. You will know the date of your second session during the weekend. [*Note from the authors: second sessions varied depending on the experiment and treatment.*]

- In today's session you will have to perform three tasks. First, you will have to choose the answer that best corresponds to your degree of agreement or disagreement with 60 statements in a questionnaire. [*Note from the authors: This was the NEO-FFI questionnaire.*]

- Secondly, you will take a pattern continuation test consisting of 40 questions and lasting 20 minutes. For each correct answer you will get 0,25€ of profit. Mistakes do not subtract. [*Note from the authors: This was the DAT-AR test.*]

- Thirdly, you will be presented with an economic task. The amount obtained in this task will be added to the amount obtained in the above-mentioned test to determine your earnings for this session. [*Note from the authors: This was the Dictator Game.*]

- The earnings from this session along with your earnings from the second session will be paid to you in cash on a confidential basis immediately upon completion of the second session.

- It is very important that you ask (by raising your hand) any questions that arise during the session. Other than those questions, any kind of communication with the other participants in the session is strictly prohibited and will get you expelled from the session.

***Instructions for Session 1 economic task***

In this game each player will play two roles: the sender and the recipient. You will be paid randomly for one of the two roles. The game consists of dividing 10€ and the sender will have an active role and the recipient will have a passive one. In the role of sender, you will have to decide which amount (with a maximum of one decimal place) of a total of 10€ you want to transfer to the recipient, who will be another person in the room, while you will keep the rest. In the role of recipient, you will not have to make any decision. You will simply be informed of the amount of euros the sender has decided to give you. You will play both roles, and the role you will be paid for will be randomly determined.

You must bear in mind that in no case will the recipient of the money that you send be the sender of the money that you receive. That is, you will play each role with a different partner.

***z-Tree Program Screen text:***

You have to decide how much of 10€ you want to transfer to another person in this room.

Decide how much you want to transfer to the other person (one decimal place separated by a dot is allowed): ____________ €
