# Supplementary material for: Gender differences in dictator giving: A high-power laboratory test
Source: PLoS One. 2025 Feb 12;20(2):e0317886. doi: 10.1371/journal.pone.0317886 (PMC11819478; doi:10.1371/journal.pone.0317886)
Supplement: Attachment — Submitted filename: Referee report_PLOS ONE_2024.docx [file pone.0317886.s002.docx]

# Referee report on *Gender differences in dictator giving: a high-power laboratory test* (Manuscript Number: PONE-D-24-05752)

This study investigates the presence of gender differences through lab experiments in the canonical game of altruism, the dictator game. As the authors note, the high number of observations allows for drawing firm conclusions in a topic on which there is not yet consensus. The authors document a significant gender difference, finding that females are more generous than males, even after accounting for a wide range of relevant control variables such as age, cognitive ability, emotions and personality traits.

This study is of high quality and makes a clear contribution to the literature, making it deserving of publication. However, there are some minor issues that should be addressed beforehand.

# Comments

I did not understand how playing the dictator game in dual roles affects the results. The authors emphasize the relevance of dual roles, but based on the description of the experiment, I did not see why this is important. Perhaps this should be made clearer.

I appreciate that this paper contributes to the debate on whether women are more generous than men. Although many studies, and perhaps even most, document a significant difference, the question remains unsettled due to the low number of observations and some influential papers (e.g., Niederle, reference 2 in the study). The authors make a strong argument for the presence of such a gender difference. Additionally, there are some recent studies (e.g., Brañas-Garza 2024, Horn et al. 2022) that use large samples to test gender differences in social preferences. This field is progressing rapidly, with studies using increasingly large sample sizes. Therefore, I suggest that the authors review the related literature from recent years to ensure that all relevant studies are included. It may also be interesting to see if these new studies with large sample sizes consistently point in the same direction.

In some cases, the authors use non-parametric tests, while in others, they use parametric ones. Using non-parametric tests consistently, which do not require a normality assumption, would make the paper more consistent and slightly more convincing.

I really liked the emotion questions. I have the impression that the authors should give them more emphasis, as they seem to be novel, easy to carry out, and potentially relevant for understanding social preferences.

# References

Brañas-Garza, P. (2024). Young teens at play: Girls are egalitarian, boys are generous. *Personality and Individual Differences*, *226*, 112703.

Horn, D., Kiss, H. J., & Lénárd, T. (2022). Gender differences in preferences of adolescents: evidence from a large-scale classroom experiment. *Journal of Economic Behavior & Organization*, *194*, 478-522.
