# Supplementary material for: Gender differences in dictator giving: A high-power laboratory test
Source: PLoS One. 2025 Feb 12;20(2):e0317886. doi: 10.1371/journal.pone.0317886 (PMC11819478; doi:10.1371/journal.pone.0317886)
Supplement: Attachment — Submitted filename: Response to the Editor and Reviewer REV2.docx [file pone.0317886.s004.docx]

PONE-D-24-05752R1

Gender differences in dictator giving: a high-power laboratory test

PLOS ONE

Dear Dr. Pavan,

Thank you for submitting your manuscript to PLOS ONE. After careful consideration, we feel that it has merit but does not fully meet PLOS ONE’s publication criteria as it currently stands. Therefore, we invite you to submit a revised version of the manuscript that addresses the points raised during the review process.

==============================

Thank you for submitting your revised manuscript. While Reviewer #2 is satisfied with the changes, Reviewer #1, while appreciative of the improved introduction and attempted structural analysis, suggests further refinements to the SEM analysis - specifically recommending a simplified mediation analysis to better demonstrate how gender effects are mediated through personality traits, reasoning ability, and emotions. As editor, I fully concur with these suggestions and request that you address these final changes in your next revision, including adding the recommended footnote about the temporal relationship between emotions and decisions.

Many thanks and alle the best,

Holger Rau

***Authors: Dear Editor, many thanks for giving us a second opportunity to improve our work. In this version, we simplified the SEM analysis and added a limitation of our measure of emotions.***

***We answer to the reviewer’s comments below (in bold italics, to facilitate reading).***

Reviewer #1: The authors addressed most of my comments sufficiently. I think they have done a great job in making their contribution clearer in the introduction and I also, appreciate that they have tried to provide a bit more structure to their analysis by including a SEM. Still, this analysis should be explained better including the assumptions made. The way it is now, I fear that it makes the contribution of the paper harder to understand rather than improving clarity. I was asking for a much simpler mediation analysis showing how much of the effect of gender on the share sent is mediated through personality traits, reasoning ability and emotions, so more of a decomposition exercise (Gelbach, 2016) as for example done by Grosch and Rau (2016). This would simplify the analysis presented quite significantly.

I think it is possible to infer what I have asked for from the model the authors provide (and they state that it can be seen in Figure 5, but not where) but it just seems unnecessarily complicated.

***Authors: Dear Reviewer, we thank you for your constructive comments and hope to have improved our work. In particular, we have simplified the SEM analysis as you suggested. We now present a simpler SEM model to test whether the gender effect is mediated by personality traits, reasoning ability and emotions. Results do not differ from our previous analysis, but we hope that they are more easily interpretable now.***

Also, the authors should at least acknowledge in a footnote that they have no way of knowing whether the emotions subjects state to have had were causing the decision or whether their reporting ex-post is affected by the decision made. For example, subjects who decided to give nothing might feel better to state that this was rational no matter what was actually driving their decision.

***Authors: Thank you for the suggestion. Since it is not possible to include footnotes in PLOS-ONE articles, we added a comment at page 18 of the paper, when describing our measure of emotions:***

***“A possible limitation of this measure is that we cannot be sure whether the emotions subjects stated to have had were causing the decision or whether their reporting ex-post was affected by the decision made.”***

References:

Kerstin Grosch, Holger A. Rau (2017). Gender differences in honesty: The role of social value orientation. Journal of Economic Psychology 62, 258-267

Gelbach, J. B. (2016). When do covariates matter? And which ones, and how much? Journal of Labor Economics 34 (2), 509–543.
